# Hand Function Recovers to Near Normal in Patients with Deep Dermal Hand Burns Treated with Enzymatic Debridement: A Prospective Cohort Study

**DOI:** 10.3390/ebj6020036

**Published:** 2025-06-12

**Authors:** Kelly Aranka Ayli Kwa, Annika Catherina Reuvers, Jorien Borst-van Breugel, Anouk Pijpe, Paul P. M. van Zuijlen, Roelf S. Breederveld, Annebeth Meij-de Vries

**Affiliations:** 1 Alliance of Dutch Burn Care, Burn Center Beverwijk, Red Cross Hospital, Vondellaan 13, 1942 LE Beverwijk, The Netherlandsjorienborst@rkz.nl (J.B.-v.B.); adevries@rkz.nl (A.M.-d.V.); 2 Department of Traumasurgery, Leiden University Medical Center, Albinusdreef 2, 2333 ZA Leiden, The Netherlands; 3 Faculty of Medicine, Vrije Universiteit Amsterdam, De Boelelaan 1105, 1081 HV Amsterdam, The Netherlands; 4 Department of Occupational Therapy, Red Cross Hospital, Vondellaan 13, 1942 LE Beverwijk, The Netherlands; 5 Plastic, Reconstructive and Hand Surgery, Amsterdam UMC Location Vrije Universiteit Amsterdam, De Boelelaan 1117, 1081 HV Amsterdam, The Netherlands; 6 Amsterdam Movement Sciences, Tissue Function and Regeneration, Boelelaan 1117, 1081 HV Amsterdam, The Netherlands; 7 Department of Plastic, Reconstructive and Hand Surgery, Red Cross Hospital, Vondellaan 13, 1942 LE Beverwijk, The Netherlands; 8 Department of Pediatric Surgery, Amsterdam UMC Location University of Amsterdam, Meibergdreef 9, 1105 AZ Amsterdam, The Netherlands; 9 Department of Surgery, Red Cross Hospital, Vondellaan 13, 1942 LE Beverwijk, The Netherlands

**Keywords:** enzymatic debridement, hand function, burns, scar quality, quality of life

## Abstract

Short- and long-term hand function was evaluated in adult patients with deep dermal and full-thickness hand burns after treatment with enzymatic debridement (NexoBrid^®^ MediWound Ltd., Yavne, Israel), assessing the results at discharge and 3, 6, and 12 months post-burn. This prospective cohort study was performed in the Burn Center in Beverwijk between March 2017 and December 2019. Hand function was assessed using Modified Kapandji Index scores, the Jebsen-Taylor Hand Function Test, and range of motion; scar quality using the Patient and Observer Scar Assessment Scale version 2.0; and quality of life using the Quick Disability Arm Shoulder Hand Questionnaire and the Canadian Occupational Performance Measure. Ten patients (14 hand burns) were included. The need for a skin graft after NexoBrid^®^ was 86%, and 50% needed additional surgical excision before skin grafting. Digits 3 and 4 achieved near-to-normal total active motion, and at least 50% of the hands achieved a normal range within the Jebsen-Taylor Hand Function Test in four items at 12 months post-burn. Scar quality and quality of life improved significantly over time. The present study can be considered as a proof-of-concept study for future clinical trials on enzymatic debridement for hand burns.

## 1. Introduction

Deep dermal and full-thickness burn wounds require surgical debridement and split skin grafting to minimize scar formation [1,2,3,4]. There are several debridement techniques, and evidence in favor of any technique is lacking [5]. The most often used methods include debridement with surgical handheld knives, hydrosurgery, and enzymatic debridement [5].

The hands are a particular area of interest when it comes to burn wounds [6]. The debridement of hand burns is often delayed in European countries to await spontaneous wound healing of the viable dermis, which allows for a more accurate assessment of the burn depth and need for excision [7]. However, this approach results in a longer healing period and increases the risk of infection. It also results in a delay in the rehabilitation of the hand.

NexoBrid^®^ (Mediwound Ltd., Yavne, Israel) is an enzymatic debriding agent consisting of proteolytic enzymes enriched in bromelain, which selectively debrides eschar in the early stage (<72 h) while leaving vital tissue unharmed [8]. Studies have shown that NexoBrid, compared to the standard of care (SOC), which usually consists of conventional tangential excision, leads to a shorter debridement time and a reduction in surgical excision and skin grafting after (hand) burns [8,9,10,11,12]. However, long-term results regarding hand function, scar quality, and quality of life (QoL) are lacking [13,14,15,16], and most older studies describe hand function after hand burns in generic terms, without the use of validated instruments [14,15,17]. Studies that evaluate hand function, scar quality, and quality of life after hand burns treated with enzymatic debridement are also limited [12,18,19].

Therefore, we performed a prospective cohort study to follow up with adults with deep dermal and full-thickness hand burns treated with enzymatic debridement, using a standardized assessment scheme measuring hand function, scar quality, and quality of life over time.

## 2. Materials and Methods

### 2.1. Study Design and Participants

Our study was a single-center prospective cohort study performed between March 2017 and December 2019 in the Burn Center of the Red Cross Hospital. The study was conducted according to the principles of the Declaration of Helsinki (Ethics Manual World Association revision 2013) and in accordance with the Medical Research Involving Human Subjects Act (WMO). The study was approved by the Medical Research Ethics Committee Noord-Holland (NL59342.094.16) and the institutional review board of the local hospital.

Eligible individuals were patients aged ≥ 18 years with a clinical diagnosis of deep dermal or mixed deep dermal and full-thickness burns of one or both hands, which were treated with enzymatic debridement (NexoBrid^®^, Mediwound Ltd., Yavne, Israel). Before enzymatic debridement, the burn wound depth was determined based on clinical assessment, and some of the patients also underwent a laser Doppler imaging scan to assess the burn wound healing potential. Patients were ineligible if they had insufficient knowledge of the Dutch language and/or if they were unlikely to comply with the requirements of the study protocol and follow-up. All patients—or their legal representatives in cases where the patient was temporarily incapacitated due to sedation and/or intubation—provided written informed consent.

### 2.2. Treatment Protocol

Patients were treated with enzymatic debridement according to the regular treatment protocol [16], which consisted of a pre-soak phase with a duration of 2–24 h with the application of gauze drenched in antibacterial solution (Prontosan^®^, B. Braun Medical B.V. Oss, The Netherlands) to prepare the wound bed, the application of enzymatic debridement for four hours, and a post-soak phase with a duration of 2–24 h with an antibacterial solution to remove the remains of enzymes and eschar. For a 180 cm^2^ burn wound (a percentage of TBSA in adults), 2 g of NexoBrid^®^ powder was used. If this was insufficient during application to achieve a firm layer of NexoBrid, an additional portion was added. Treatment after enzymatic debridement varied between patients depending on dermal preservation. In cases of complete dermal preservation, e.g., no full-thickness spots, an allograft or Suprathel^®^ (Polymedics Innovations GmbH, Denkendorf, Deutschland) was used. If full-thickness spots were present, then an allograft was applied. After the NexoBrid^®^ procedure, all patients received an intrinsic plus splint for the day and night that stabilized the wrist in an extended position of 25° degrees and the MCP joints in a flexed position of 70° degrees. After 3 to 7 days post-NXB, the patients started to mobilize their hand(s) under the guidance of the occupational therapist and followed daily instructions/hand tasks during admission. If a patient was intubated, the occupational therapist provided daily motion of the burned hands. Re-epithelialization was allowed to proceed for approximately 4–5 weeks, and, as soon as re-epithelization had been completed, patients received pressure garments to decrease erythema and burn scars [13]. In cases where re-epithelization stagnated or in cases of larger full-thickness burn spots, split skin grafting was performed. Conservatively treated burns were treated until wound closure using a combination of antibacterial solutions (e.g., Bactroban^®^, GlaxoSmithKline B.V. Amersfoort, The Netherlands or Fucidin^®^, LEO Laboratories Ltd., Dublin, Ireland), based on the patients’ culture swabs. All culture swabs were assessed by a microbiologist.

### 2.3. Clinical Characteristics and Outcomes

These include age, sex, burn etiology, % total body surface area (TBSA), % body surface area (BSA) burned of the hands (BSA), the need for escharotomy, surgical excision, autografting, the percentage TBSA for the autografting of their burned hands, the time to reach wound healing of their hands (defined as re-epithelialization > 95%), bacterial colonization, the length of hospital stay (LOS), the dominance of the hand, comorbidities, smoking habits, return to work, and hand therapy.

### 2.4. Study Outcomes

#### 2.4.1. Hand Function

Hand function was measured at discharge and at 3, 6, and 12 months post-burn by an occupational hand therapist, trained and specialized in the treatment of burn patients and hands [JB-vB] using a standardized assessment scheme. This scheme was developed by specialists in the field of (hand) burn care and based on reliable and valid measurement instruments. The scheme consisted of the following assessments.

- Jebsen-Taylor Hand Function test (JTHFT): The JTHFT is a standardized and objective test with 7 items representative of various hand activities, which include (1) writing a short sentence, (2) turning over 3 × 5-inch index cards, (3) picking up small objects (paperclip, coin), (4) stacking checkers, (5) simulating eating, (6) picking up large light objects (empty cans), and (7) picking up large heavy objects (full cans). The activity is measured by the time that it takes to complete the activity and is either within a normal range (1 = yes) or not within a normal range (0 = no) [20].
- Range of Motion (ROM) (goniometry): Goniometry is used to measure the passive (PROM) and active (AROM) ROM of the wrist or finger joints. The AROM angles for each finger are described using the total active motion (TAM) score, which is the sum of the MCP, IP, PIP, and DIP joints for each digit minus the extension deficit of the measured digit [21]. A TAM of 260° was considered normal for digits 2–5. The lower arm during the measurement was placed in a neutral, flexed position of 90° and the wrist in an extended position of 20°. The AROM angles were assessed in a composite manner [22].
- Modified Kapandji Index (MKI) [23]: The MKI is a combined score from three tests: (1) a thumb opposition test, by scoring from 0 (impossible to do) to 10 (completely accomplished); (2) a finger flexion test; and (3) a flat hand/extension finger test, by scoring from 0 (impossible to do) to 5 (completely accomplished). The maximum sum score is 35 points, indicating optimal function. This assessment was only performed if the patient was fully conscious.

#### 2.4.2. Scar Quality

Scar quality was measured using the Patient and Observer Scar Assessment Scale, version 2.0 (POSAS 2.0) [24] (www.posas.nl accessed on 1 March 2017), at 3, 6, and 12 months post-burn. The POSAS consists of a patient and an observer scale. The patient scores include pain, pruritus, color, thickness, relief, pliability, and overall opinion, on a scale ranging from 1 (“no, not at all”) to 10 (“yes, very much”). The observer scores include vascularization, pigmentation, thickness, surface roughness, pliability, surface area, and overall opinion, on a scale ranging from 1 (“normal skin”) to 10 (“worst scar imaginable”). Two different trained observers completed the observer part of the POSAS, and the scoring of their items was averaged [25].

#### 2.4.3. Quality of Life

Quality of life was measured at 3, 6, and 12 months post-burn by the following validated questionnaires.

- Quick Shortened Disability Arm Shoulder Hand (Q-DASH) Questionnaire: The Q-DASH is a shortened version of the DASH, which is a patient self-rated questionnaire that is specific to the function of the upper limb extremity, and has a scale from 1 (“no difficulty”) to 5 (“impossible to carry out”); it provides a minimum total score of 0 (best) and a maximum score of 100 (worst) [26]. Patients also provided a Q-DASH questionnaire filled in as if the situation was pre-burn.
- Canadian Occupational Performance Measure (COPM): The COPM is a tool used by occupational therapists to conduct a semi-structured interview to identify issues in areas of self-care, productivity, and leisure for individual patients. Each of these problems is rated based on performance and satisfaction on a scale from 0 (worst) to 10 (best). Mean scores were calculated per patient, independently of the number of problems that they reported [27].

### 2.5. Statistical Analysis

Continuous data were presented as the mean and standard deviation (SD, ±) in the case of parametric data and as the median and interquartile range (IQR) in the case of non-parametric data. The Friedman test (continuous data, categorical variables) was used to assess hand function, scar quality, and QoL over time. In cases of statistically significant differences over time, a post hoc analysis with either the Wilcoxon (values in >2 scales) or McNemar test (dichotomous values) was conducted to evaluate the changes between the time points. The *p*-value of < 0.05 was taken as a threshold for statistical significance. In cases where a post hoc analysis was conducted, a Bonferroni correction was applied (*p* = 0.05 divided by the number of tests). The *p*-values provided in the Results section are the applied significance levels corresponding to the test performed.

## 3. Results

### 3.1. Clinical Characteristics

**Figure 1:**
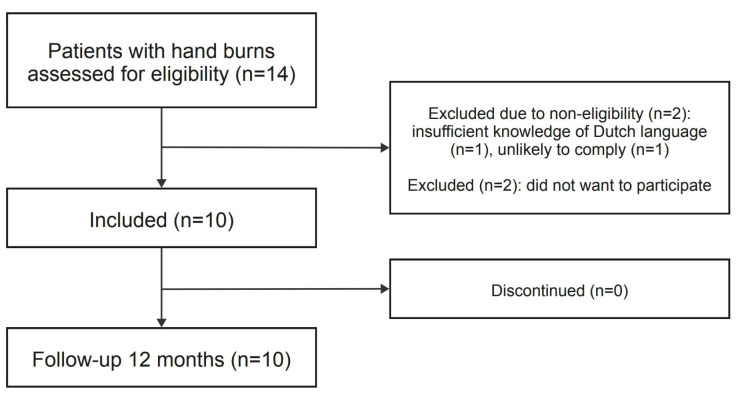
Flowchart.

**Figure 2:**
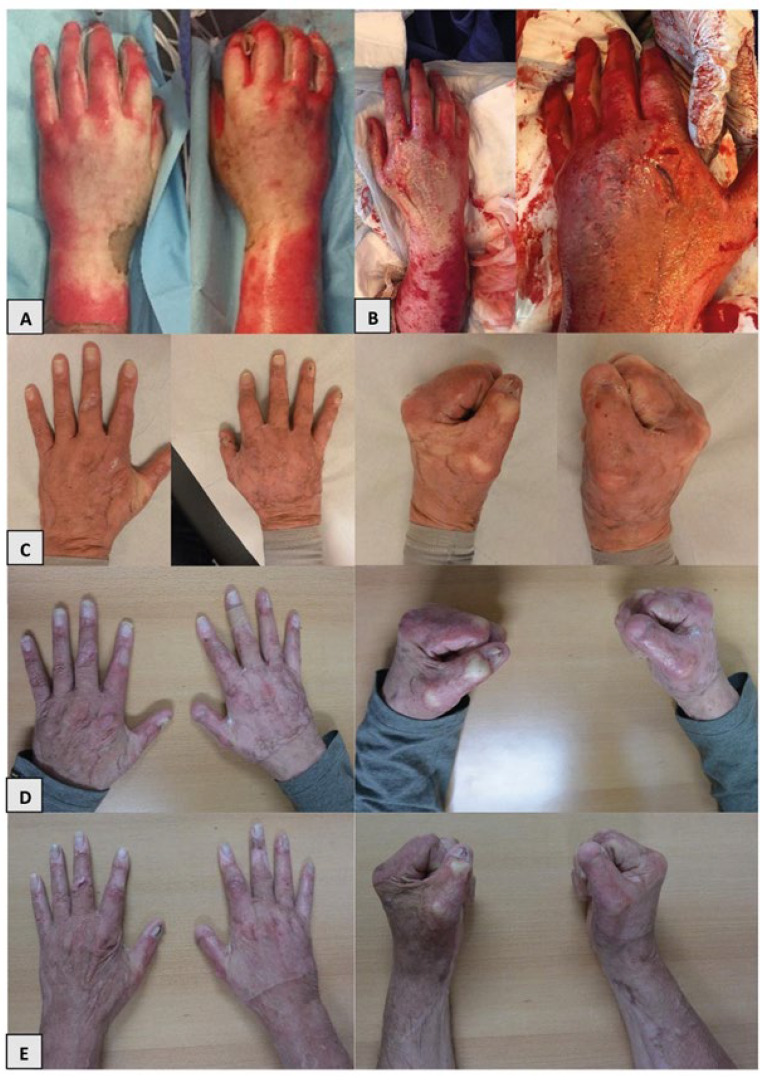
Evaluation of the hands after NexoBrid^®^ treatment. (**A**) = flame burn; (**B**) = post-enzymatic debridement; (**C**) = 3 months post-burn; (**D**) = 6 months post-burn; (**E**) = 12 months post-burn.

Of the 14 available patients, two patients were excluded due to non-eligibility and another two patients declined to participate. We evaluated 14 consecutive hands of 10 patients between March 2017 and December 2019 (Figure 1). The patient group consisted of nine men and one woman, and they had a mean age of 56.3 ± 12.5 years (Table 1). Eight patients had both hands burned, among which, in four patients, both hands were treated with enzymatic debridement, leading to the inclusion of 14 enzymatically treated hands. All digits of these included hands were affected. The burns in one patient (both hands) were limited to the dorsal side of the hands. The other nine patients had burns on both the dorsal and volar sides of their hand(s). Nine patients received hand therapy directly upon admission. The duration of pressure garment use had a median of 8 months. Silicon therapy was administered in eight patients, with a median of 6 months. None of the patients received corticosteroid treatment (Figure 2).

### 3.2. Clinical Outcomes

Five patients (seven hands) with a TBSA of the enzymatically treated hand of 1.5% (IQR 1.5–2.0) required additional excision of 1.0% (IQR 1.0–1.5) TBSA located on the treated hand with the Versajet Hydrosurgery System (Smith & Nephew, Memphis, TN, USA) or a Weck blade at a median of 15 (IQR 1–21) days after enzymatic debridement to produce a vital wound bed so that skin grafting could take place. In four hands (three patients), the Versajet was used. Tangential excision with the Versajet and a Weck blade was performed in one patient (two hands). In one patient (one hand), the method of surgical debridement was not documented. In three patients (five hands) with a TBSA of the enzymatically treated hand of 2.0% (IQR 1.3–2.5), additional skin grafting without excision was needed of 0.9% (IQR 0.6–1.5) TBSA located on the treated hand after a median of 16 (IQR 2–24) days after enzymatic debridement. One of the patients received an Integra^®^ (LifeSciences, Plainsboro, NJ, USA) dermal regeneration template on both hands as the burn wound was a full-thickness wound and required arthrodesis of the IP joint. The median time to wound healing of the enzymatically treated hands was 31.0 (IQR 24.0–39.0) days.

### 3.3. Return to Work

Seven out of ten patients were working at the time of their burns. At 12 months, all seven patients had returned to work, among which four reported a reduction in the hours worked compared to the pre-burn period. All patients kept the same profession, albeit in an adjusted setting.

### 3.4. Study Outcomes

#### 3.4.1. Hand Function

**Figure 3:**
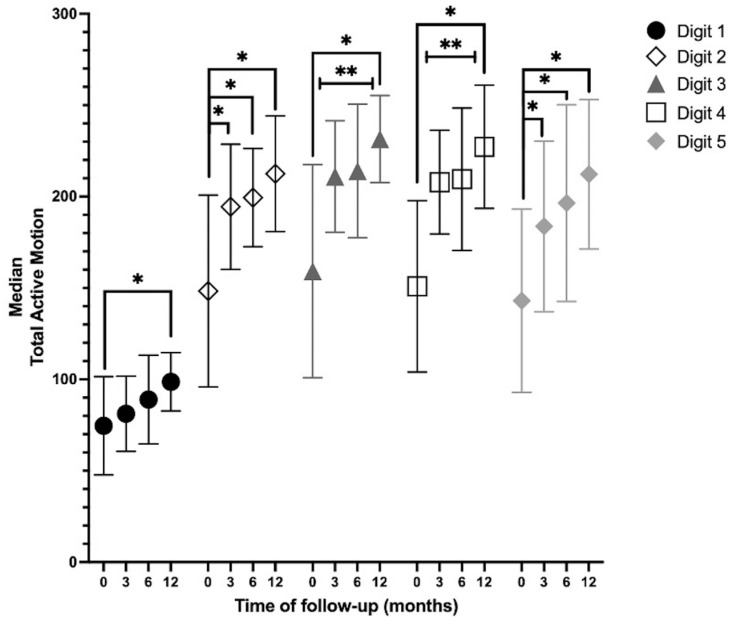
Goniometry; median TAM over time. TAM = total active motion; 260° is considered normal. Friedman test, post hoc analysis, Wilcoxon signed-rank test, *p* < 0.0083. * Significant increase between the indicated months. ** Significant increase between every month, except between 3 and 6 months.

**Figure 4:**
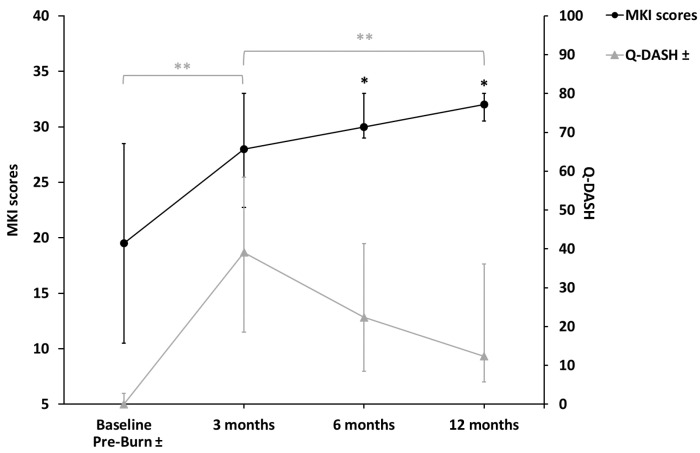
MKI scores and Q-DASH. MKI = Modified Kapandji Index. Q-DASH = Quick (shortened) Disability Arm Shoulder Hand Questionnaire. Friedman test, post hoc analysis, Wilcoxon signed-rank test. ● *compared to baseline; *p* < 0.0083. 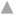 ** significant for Q-DASH; *p* < 0.017.

- *JTHFT*: Cochran’s *Q* test determined that there was a statistically significant difference in the outcome of the test over time in picking up large light objects (n = 14, X^2^(2) = 7.60, *p* = 0.022) and picking up large heavy objects (n = 14, X^2^(2) = 6.50, *p* = 0.039). The post hoc analysis revealed no statistically significant differences between the time points. At 12 months, 11 (78.6%), 8 (57.1%), 8 (57,1%), and 7 (50%) hands achieved a normal range in items 1, 2, 6, and 7, respectively.
- *AROM:* Digits 2, 3, and 5 showed an increase in the median TAM during all measurements. Digits 1 and 4 showed an increase in the median TAM during all measurements, except for the measurements between 3 months and 6 months. In digits 3 and 4, there was a statistically significant increase between baseline and 3, 6, and 12 months; between 3 months and 12 months; and between 6 months and 12 months. In digits 2 and 5, there was a statistically significant increase in the TAM over time between baseline and 3, 6, and 12 months. At 12 months, the TAMs of digits 3 and 4 returned to near normal (260°) (Figure 3).
- *MKI:* There was a statistically significant increase over time between baseline and 6 months (*p* = 0.008) and between baseline and 12 months (*p* = 0.002) (Figure 4).

#### 3.4.2. Scar Quality

**Figure 5:**
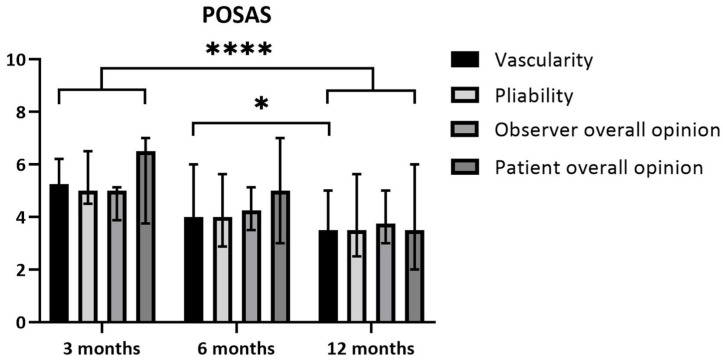
POSAS scores over time. POSAS = Patient and Observer Scar Assessment Scale. Friedman test, * *p* < 0.05; post hoc analysis, Wilcoxon signed-rank test, *p* < 0.017, **** indicates statistical significance for all four items mentioned.

- *Patient scores:* The overall opinions of the patients regarding their scars 3 months after the burn yielded a median of 6.5 (IQR 3.8–7.0) and median of 3.5 (IQR 2.0–6.0) after 12 months. This improvement did not reach statistical significance based on the corrected threshold *p*-value (*p* = 0.029; threshold *p* < 0.017) (Figure 5).
- *Observer scores:* The overall opinion of the observer regarding the scars 3 months after the burn yielded a median of 5.0 (IQR 3.9–5.1) and a median of 3.8 (IQR 3.0–5.0) after 12 months. This difference over time was statistically significant, with *p* = 0.011. The scores for pliability improved between 3 and 12 months, with a median of 5.0 (IQR 4.5–6.5) at 3 months and a median of 3.5 (IQR 2.5–5.6) at 12 months. This difference was statistically significant (*p* = 0.011). The scores for vascularity showed a median of 5.3 (IQR 3.9–6.1) at 3 months and a median of 3.5 (IQR 1.9–5.0) at 12 months (*p* = 0.002). Vascularity also improved significantly between 6 and 12 months post-burn. The median at 6 months was 4.0 (IQR 3.8–6.0) and the median at 12 months was 3.5 (IQR 1.9–5.0) (*p* = 0.009) (Figure 5).

#### 3.4.3. Quality of Life

- *Q-DASH:* There was a statistically significant increase (*p* = 0.005) between the pre-burn period (median 0.0, IQR 0.0–2.8) and 3 months (median 39.1, IQR 18.6–58.4) and a reduction between 3 months and 12 months (median 12.3, IQR 5.8–36.1) (*p* = 0.005) (Figure 4).
- *COPM:* There was a statistically significant increase (*p* = 0.005) in the performance scores between 3 months (median 6.1, IQR 3.8–7.4) and 12 months (median 8.8, IQR 7.9–9.8) and between 6 months (median 7.0, IQR 5.0–8.5) and 12 months (*p* = 0.005). There was a statistically significant increase (*p* = 0.011) in the satisfaction scores between 3 months (median 5.5, IQR 1.5–8.0) and 6 months (median 8.0, IQR 5.5–8.6) and between 3 and 12 months (median 8.3, IQR 7.6–9.9) (*p* = 0.005), but not between 6 and 12 months (*p* = 0.021).

## 4. Discussion

Our study showed a significant improvement in hand function after hand burns were treated with enzymatic debridement (NexoBrid^®^). Digits 3 and 4 showed a significant increase in TAM during all time measurements, except between 3 months and 6 months, and achieved near-to-normal TAM at 12 months post-burn. At least half of the included hands achieved a normal range on the JTHFT in four items at 12 months post-burn. Regarding scar quality, both patients and observers showed an improvement in the overall opinion on the POSAS 2.0 between 3 months and 12 months. In our study, half of the treated hands needed additional surgery, and 12 hands required a skin graft after enzymatic debridement.

Hand function in our study was assessed using the JTHFT, the AROM, and the MKI, which measure the functional outcomes of the hand and fingers. The JTHFT showed no significant improvement over time. Despite this, ≥ seven hands achieved a normal range in four of the seven items. With regard to enzymatic debridement, only some studies have looked solely at hand burns and hand function [9,12,18,19,28,29,30,31,32]. However, studies assessing hand function in patients with partial to deep hand burns after treatment with NexoBrid^®^ are scarce. The study by Malsagova et al. (2024) [32] examined hand function in patients with superficial partial-thickness, deep partial-thickness, and full-thickness hand burns who received either conservative or surgical treatment after enzymatic debridement with NexoBrid^®^, with a mean follow-up period of 31 months post-burn. They also described a normal range of motion in most of the included hands. The study by Corrales-Benítez et al. (2022) also assessed hand function using a goniometer in patients with deep partial-thickness hand burns after treatment with enzymatic debridement [18].

The recovery pattern of our patients regarding the TAM corresponds with the results of Ghalayini et al. (2019) [33] Their study also described a similarly slow recovery pattern between 3 and 6 months.

Besides the improvement in the POSAS 2.0 item overall opinion, we found a significant decrease in the observer score for the overall opinion, vascularity, and pliability over time. At 12 months, the overall opinion on scar quality of the observer part of the POSAS in our study was slightly higher compared to the study of Heitzmann et al. (2024) [19]. This was also the case in the items vascularity and pliability, in which patients reported a more positive effect in the study of Heitzmann et al. (2024) [19]. A possible explanation as to why the patients did not report a difference in color over time is as follows: while observers are asked to report specifically on vascularity (redness), patients are asked to assess the color of their scars. Over time, deep burn scars often develop from red (due to increased vascularity) to hyper- or hypopigmentation. Thus, the patients possibly scored highly on color due to the change in pigmentation over time.

The application of NexoBrid^®^ in burn wounds, especially in hands, is increasing due to its capacity for selective debridement, thereby preserving the viable dermis, which reduces the need for surgery and autografting [8,10,12,34,35]. During this study, we awaited re-epithelialization for approximately 4–5 weeks. Skin grafting was performed if re-epithelialization stagnated or the wound bed was deteriorating or in cases of larger, full-thickness burn spots. However, we are aware that, nowadays, the decision regarding skin grafting can be made almost immediately after treatment with NexoBrid, due to improved clinical insights [35].

As mentioned earlier, in our study, 50% (7 out of 14 hands) required additional excision, and the need for skin grafting in the treated hands was 86% (12 out of 14 hands). The need for additional excision is not uncommon after enzymatic debridement and might in part be explained by the requirement for skin grafting in cases of deep dermal and/or full-thickness burn wounds. Several studies have described the need for additional excision after enzymatic debridement [12,29]. In the studies by Dadras et al. (2020) and Cordts et al. (2016), 28/52 hands (53.8%) and 6/16 patients (46.2%) with deep partial- to full-thickness hand burns treated with enzymatic debridement required an additional skin graft, respectively [10,30]. However, 31 patients with partial-thickness to deep hand burns did not need an additional skin graft after ED in the study of Heitzmann et al. (2024) [19]. On the other hand, deep partial-thickness hand burns contain more viable dermis compared to the patients that we included with deep dermal and full-thickness hand burns.

Despite our results regarding the need for additional surgical excision and a skin graft in deep dermal and full-thickness hand burns, enzymatic debridement reduces the surface area of the excision or skin graft by sparing vital tissue. This makes the treatment possibly superior compared to tangential excision in functional parts of the body, such as the hands [19,29,31,36].

Our study has several strengths. The debridement of deep dermal burns has so far mainly been focused on clinical outcome parameters, e.g., the time to wound healing and time to debridement, and little is known about long-term scar quality as an outcome of the debridement technique—specifically in enzymatic debridement [5]. Moreover, studies on hand function after burn wounds are lacking. The definition of good hand function does not solely rely on an adequate range of motion, which is a limited measurement because it is not a reflection of functional ability [37,38]. This is why we chose to assess several domains of hand function, scar quality, and QoL using multiple validated instruments. Moreover, all measurements were completed by the same occupational hand therapist and we reduced the immobilization time [39].

Our study has some limitations. Firstly, we were not able to perform a comparative or randomized study in which we compared the results with those for patients treated with the standard of care. We did not wish to withhold patients with deep hand burns from treatment with enzymatic debridement, and patients with deep dermal hand burns are relatively limited. Secondly, the heterogeneity of our patient group was a limitation. Thirdly, we did not consider other patient factors that might have influenced the QoL outcome.

The present study may be considered as a proof-of-concept study on enzymatic debridement for hand burns and a feasibility study on a comprehensive outcome assessment protocol. A next step could be a multi-center randomized controlled trial or a trial within cohorts (TwiCs) study.

## 5. Conclusions

In conclusion, hand function, scar quality, and quality of life in patients with deep dermal hand burns treated with enzymatic debridement improved significantly over time. Patients had a significant improvement in hand function at 12 months post-burn, especially in digits 3 and 4, and all employed patients returned to their previous professions.

## Figures and Tables

**Table 1:** Patient and clinical characteristics.

General (n = 10, 14 Enzymatically Treated Hands)	
Male (n)	9
Age (years)	56.3 ± 12.5
Smoking (yes, n)	3
Comorbidities (yes, n):	
Diabetes mellitus	1
Cardiovascular disease	2
Cause of burn:	
Flame (n)	9
Scald (n)	1
Right hand dominance (n)	9
TBSA * burned total (%)	11.0 ± 8.1
Time to wound healing (days)	35.1 ± 12.6
Length of hospital stay (days)	25.3 ± 15.6
Enzymatically Treated Hands (n = 14)	
TBSA * burned (%)	1.8, 1.5–2.5
TBSA * burned 2nd degree (%)	1.5, 1.0–2.5
TBSA * burned 3rd degree (%)	0, 0–1.0
TBSA * excised (%)	0.3, 0–1.3
TBSA * skin grafted (%)	1.0 ± 0.6
TBSA^*^ skin grafted in percentage of TBSA burned (%)	57.6 ± 31.7
Time to wound healing (days)	31.0, 24.0–39.0
Wound colonization pathogenic bacteria (n)	6

Values are presented as mean with standard deviation (±) in case of parametric data and as median with interquartile range in case of non-parametric data. * TBSA = total burn surface area.

## Data Availability

The data presented in this study are available on request from the corresponding author due to privacy reasons.

## Abbreviations

The following abbreviations are used in this manuscript:

AE	Adverse Event
AROM	Active Range of Motion
COPM	Canadian Occupational Performance Measure
ED	Enzymatic Debridement
JTHFT	Jebsen-Taylor Hand Function Test
LOS	Length of Hospital Stay
MKI	Modified Kapandji Index
POSAS	Patient and Observer Scar Assessment Scale
Q-DASH	Quick Shortened Disability Arm Shoulder Hand Questionnaire
QOL	Quality of Life
ROM	Range of Motion
SOC	Standard of Care
TAM	Total Active Motion
TBSA	Total Body Surface Area
